# Evaluation of the Effect of Local Anesthesia for Dental Treatment on Physiological Parameters and Recovery Outcomes in Children Undergoing Dental Treatment Under General Anesthesia

**DOI:** 10.1155/ijod/4297430

**Published:** 2025-08-30

**Authors:** Ali Baghalian, Bita Malekianzadeh, Sara Ghadimi, Mohammad Hassan Hamrah

**Affiliations:** ^1^Department of Pediatric Dentistry, School of Dentistry, Tehran University of Medical Sciences, Tehran, Iran; ^2^Department of Anesthesiology, School of Medicine, Tehran University of Medical Sciences, Tehran, Iran; ^3^Department of Public Health and Health Systems, Nagoya University Graduate School of Medicine, Nagoya, Japan

**Keywords:** general anesthesia, local anesthesia, pain, pediatric dentistry

## Abstract

**Objectives:** The purpose of this study was to evaluate the effect of local anesthesia (LA) for dental treatment of children under general anesthesia (GA) on physiologic parameters of patients and recovery of the patients. This information is important because currently, no guidelines exist to define the usage of local anesthetics in dental cases under GA.

**Methods:** This study was designed as a double-blinded randomized clinical trial. Intraoperatively, physiologic parameters, including heart rate, mean arterial pressure (MAP), arterial oxygen saturation, and end-tidal carbon dioxide (ETCO_2_) were observed during points of potential stimulation, and the corresponding time was recorded from the monitor of the operation room. The Aldrete scoring system was then recorded for all the included participants.

**Results:** There were 34 children (55.9% boys, 44.1% girls, age 2–7 years) for whom the dental treatment was done under GA in the study. The mean age was 4.08 ± 1.31 years. ETCO_2_ was significantly lower in the group that received LA than in those that did not (*p*  < 0.05). Aldrete's score in the group that received LA was significantly higher than that did not (9 vs. 9.47 ± 0.51, *p* = 0.002).

**Conclusion:** In conclusion, we found LA provides better intraoperative vital sign stability, particularly regarding heart rate and ETCO_2_. Also, patients who did not receive LA had statistically significantly higher ETCO_2_ than those patients who received LA.

**Trial Registration:** Iranian Registry of Clinical Trials: IRCT20220425054651N1

## 1. Introduction

Early childhood caries (ECC) remains among the most prevalent chronic conditions affecting children globally [1]. It is a public health problem affecting children worldwide [2]. ECC significantly impacts children's development, speech, overall health, and self-esteem, thereby diminishing their quality of life [3, 4].

One of the most critical challenges in pediatric dentistry is behavior management. The behavior of pediatric patients during dental procedures plays a crucial role in determining the success of the treatment [4]. Managing children is particularly challenging for dentists due to behavioral management difficulties in young children [5]. Thus, the American Academy of Pediatric Dentists has published guidelines for behavior management methods in children with dental problems, including Tell-Show-Do, voice control, positive reinforcement, distraction, and nonverbal communication. Also listed are the hand-over-mouth technique and physical restraint. The last three methods comprise pharmacological interventions such as conscious sedation, nitrous oxide (N_2_O), and general anesthesia (GA) [4].

GA induces a state of drug-induced unconsciousness, rendering the patient unresponsive to even painful stimuli, including surgical procedures. Recognized as an advanced behavior management method by the American Academy of Pediatric Dentistry (AAPD), it facilitates extensive dental treatments in young and precooperative children, ensuring care is provided in a safe, minimally restrictive, effective, and comfortable manner [6]. Its advantages in dentistry include providing immobility, amnesia, sedation, and analgesia [7]. Enabling significant dental procedures to be completed in a single session with minimal discomfort or memory of the experience [8]. Local anesthesia (LA), on the other hand, temporarily disrupts neural conduction by blocking sodium ion influx through neuronal membrane channels [7]. Widely used in both medicine and dentistry, it prevents pain during procedures and helps manage postoperative discomfort [9, 10]. Unlike GA, LA allows for a temporary loss of sensation in a specific area without affecting the patient's consciousness [11].

Local anesthetics are categorized into two main chemical classes: amides and esters [12]. Ester anesthetics, such as procaine and benzocaine, differ from amide anesthetics, which include lidocaine, articaine, mepivacaine, bupivacaine, and prilocaine [13]. Although many anesthetics are used in dentistry, a smaller selection is used in pediatrics [14]. A study by Brickhouse et al. [8] evaluated local anesthetic usage among providers caring for children. It noted that despite an increase in articaine usage, lidocaine with epinephrine is still the preferred dental local anesthetic for use in children [14]. While LA is universally employed for both sedated and nonsedated pediatric patients in dental offices, its application during dental procedures under GA is less clearly defined [15].

This study aims to evaluate the impact of LA during dental treatments performed under GA on children's physiological parameters and recovery outcomes. This research is significant as there are currently no established guidelines defining the use of local anesthetics in dental procedures conducted under GA [16].

## 2. Materials and Methods

This study was designed as a double-blinded, randomized clinical trial. The research was performed under GA at the Department of Pediatric Dentistry, School of Dentistry, Tehran University of Medical Sciences, Tehran, Iran. The Human Ethics Review Committee of the School of Dentistry, Tehran University of Medical Sciences, approved the research protocol (IR.tums.dentistry.rec.1400.159), and it was also registered in clinical trials (IRCT20220425054651N1). The sample size was calculated using the formula for comparing means in two independent groups. By considering a Type I error rate of 0.05 (*α* = 0.05) and a power of 80% (*β* = 0.20), the calculation was based on the following parameters:

  $$\begin{array}{c} n=\frac{{\left(z_{1-\alpha /2}+z_{1-\beta }\right)}^{2}\left(\delta_{1^{2}}+\delta_{2^{2}}\right)}{{\left(\mu_{1}-\mu_{2}\right)}^{2}}, \end{array}$$

where *Z*_1_−*α*/2 = 1.96 (corresponding to *α* = 0.05), *Z*_1_−*β* = 0.84 (corresponding to 80% power), *σ*_1_ and *σ*_2_ are the standard deviations for the two groups, derived from the study by El Batawi [17], and μ1−μ2 is the mean difference between the two groups, derived from El Batawi [17].

Using the means and standard deviations reported in El Batawi [17] the required sample size for each group was calculated to be 17 children. All patients included were healthy children (ASA I or ASA II), aged 2–7 years, without any history of behavioral issues, and planned to undergo dental rehabilitation, including prefabricated crowns and pulp therapy. All patients were included in a randomized parallel clinical trial design study. Informed consents were obtained from the parents. All the patients were previously identified as requiring GA for their dental care due to precooperative/uncooperative behavior and/or scope of treatment. All patients were assigned a participant number used to randomize participants and identify patient information for data collection. Participant numbers were used to randomize patients, assigning them to LA (experimental) or no LA treatment groups (control group). Randomized groups were assigned participant numbers, reviewed by the dental surgeon. However, to maintain blinding, the surgeon was not informed of the patient's group allocation. All other research team members, including the anesthesiologist, as well as parents or guardians, were blinded to the participants' treatment groups. The anesthetic syringes were prepared by a separate research assistant to ensure that both the surgeon and the anesthesiologist remained unaware of the intervention group.

The standard LA used for the experimental group was 2% lidocaine with 1:100,000 epinephrine (Daru Pakhsh Pharmaceutical Mfg. Co., Tehran, Iran). Lidocaine was selected due to its well-established safety profile and widespread use in pediatric dentistry. The total amount of LA given was noted, with no patient exceeding 4.4 mg/kg. Since for inferior alveolar nerve blocks there is a chance of unsuccessful block, all the interventions in this study were focused on maxillary teeth, which have a high chance of anesthetic success by infiltration. The two most painful procedures in pediatric dentistry, including extraction and pulpotomy, were selected to evaluate the effect of LA on physiologic parameters. After infiltration of the local anesthetic, in order to give time for the maximum effect of local anesthetic, prophylaxis was performed. After prophylaxis, the first procedure began. The dentist announced when each evaluated procedure would be performed. The data recorder wrote the time of the procedure and recorded the vital signs after a 3-min interval following the start of the painful procedure. Physiological parameters were recorded before the procedure, immediately upon entering the pulp chamber, and after removing the coronal pulp. The general anesthetic intervention was noted simply as “yes” or “no” for each procedure. The patient received propofol in 10 mg boluses when the intervention was needed. The anesthesiologist intervened when any of the following criteria were met: patient movement; breath holding; vital sign increases 20% above baseline; or end-tidal carbon dioxide (ETCO_2_) less than 40 mmHg.

In both groups, vital signs, including heart rate, mean arterial pressure (MAP), arterial oxygen saturation, ETCO_2_, and corresponding time were recorded from the monitor of the operation room. The Aldrete scoring system was then recorded for all the participants. Aldrete's scoring system is a commonly used scale for determining when postsurgical patients can be safely discharged from the postanesthesia care unit, generally to a second-stage recovery area, hospital ward, or home [18].

IBM SPSS software version 22 was used for statistical analysis. The qualitative and quantitative data were respectively reported and described as means (standard deviations) and numbers (%). The 2 groups were compared using Chi-square or Fisher's exact test if appropriate for categorical variables, and independent sample *t*-test (Mann–Whitney *U* test) for continuous variables. The changes in MAP and heart rate were compared between treatment and control groups using repeated-measures ANOVA. The *p*-value less than 0.05 was set as the significance level.

## 3. Results

There were 34 children (55.9% boys, 44.1% girls, age 2–7 years) for whom the dental treatment was done under GA in the study. The mean age was 4.08 ± 1.31 years, and half of the participants were less than 4 years old. The study population was equally divided into two intervention groups (with and without LA) (17 patients in each group). The two groups were similar in terms of age and gender distribution. ETCO_2_ was significantly lower in the group that received LA than in those that did not (36.17 ± 0.46 vs. 37.41 ± 1.97, *p* = 0.046). Aldrete's score in the group that received LA was significantly higher than those who did not (9 vs. 9.47 ± 0.51, *p* = 0.001). (Table 1) The characteristics of the two groups are compared. HR and MAP values at individual time points did not differ significantly between groups (*p*  > 0.05). However, repeated measures ANOVA revealed significant HR changes over time within both groups (*p*  < 0.001), indicating intraoperative differences not captured by single time-point comparisons. For MAP, the assumption of sphericity was violated (Mauchly's test *p*  < 0.001), so the Greenhouse–Geisser correction was applied.

Repeated measures ANOVA test was used to compare MAP and HR changes between the two groups with and without LA.  Table 2 shows the MAP values immediately after the start of treatment, the moment of entering the pulp chamber, and the end of removing the coronal pulp in the two study groups.

Table 3 results indicate that Mauchly's test for MAP showed that the assumption of covariance is not homogeneous (*p*  < 0.001). Accordingly, a within-subjects effect analysis was reported based on the Greenhouse–Geisser test.

Also, due to the interactive effect between LA and HR, HR changes were studied separately in the two study groups. Accordingly, HR changes in both groups were statistically significant (Table 4).

## 4. Discussion

This study aimed to determine the effect of LA on the physiological parameters and recovery outcomes of children undergoing dental treatment under GA, with a particular focus on pulpotomy procedures. Both intraoperative physiological parameters and patient recovery were evaluated. We observed that patients who received preemptive LA exhibited lower heart rates and MAP compared to those who did not. Similarly, El Batawi [17] reported significantly higher heart rates during procedures such as dentinal tissue cutting, tooth extraction, and pulpotomy in patients who had not received LA.

The increased heart rate observed during potentially painful procedures may indicate intraoperative patient pain. However, no additional intervention from the anesthesiologist was required at the levels recorded in this study. Similarly, Watts et al. [19] (2009) found that LA minimized intraoperative physiological fluctuations, including heart rate and blood pressure, in children undergoing dental procedures under GA. Their study demonstrated that LA reduced the need for anesthesiologist intervention by stabilizing physiological responses, which supports our findings [19].

Patients who did not receive LA exhibited significantly higher ETCO_2_ levels compared to those who did, suggesting that these patients were more comfortable. This trend implies that the patients were taking fewer, more relaxed breaths and emphasizes the direct link between ETCO_2_ and cardiac output, which is primarily dependent on heart rate in children. This finding is consistent with Watts et al. [19], who reported that LA reduced intraoperative physiological fluctuations and minimized the need for anesthesiologist intervention during dental rehabilitation under GA.

Similar findings were reported by Watts et al. [19], who observed elevated ETCO_2_ during dental extractions and pulpotomies in patients lacking preemptive analgesia or LA, and by El Batawi [17], who noted increased ETCO_2_ during these procedures in patients without LA. No statistically significant differences were found in respiratory rate, systolic blood pressure, or diastolic blood pressure between groups. This consistency with Watts et al. [19] further underscores the lack of significant intraoperative vital sign changes aside from ETCO_2_ differences between the LA and no-LA groups. The increased heart rate and ETCO_2_ during potentially painful procedures may serve as indicators of intraoperative patient pain. However, at the levels observed, no additional anesthesiologist intervention was required. The benefits of LA in dental procedures under GA remain unclear and warrant further research to determine its role in preventing central sensitization and managing postoperative pain in such cases.

The final aspect evaluated in this study was the intraoperative stimulation caused by crown seating and pulp therapy. Significant differences were noted in all physiological parameters during these procedures, particularly during the second phase of data collection when entering the pulp chamber without LA. This phase showed a marked increase in physiological parameters, underscoring the potentially stimulating nature of this treatment. In clinical practice, this finding highlights the importance of ensuring patient comfort, particularly for both sedated and nonsedated patients, by providing adequate LA during such procedures.

We used the Aldrete score as a criterion for discharging patients. This scoring system evaluates respiration patterns, consciousness level, SPO2 levels, blood pressure, and physical activity (motor function). A score of 8 or higher out of 10 was deemed sufficient for discharge [18]. Our results revealed a significant difference in Aldrete scores between the LA and non-LA groups. Patients in the LA group demonstrated a higher average Aldrete score (9 vs. 9.47 ± 0.51, *p* = 0.001), indicating improved postoperative recovery. This resonates with Sarapultseva and Sarapultsev [20], who reported improved recovery scores in patients receiving adjunct analgesia under GA, particularly when muscle relaxants were used. However, the impact of LA on long-term postoperative pain remains inconclusive. Our results showed some benefit, but not to a statistically significant level. This finding mirrors conclusions from a Cochrane review, which reported that while LA may reduce intraoperative pain, its role in postoperative analgesia under GA remains uncertain.

These results have clinical implications. The targeted pain relief provided by LA can reduce the need for systemic analgesics, contributing to better recovery outcomes. Additionally, the intraoperative stability in vital signs observed with LA administration highlights the benefits of its use at the start of treatment in cases under GA. This approach offers dual advantages: maintaining intraoperative physiologic stability and achieving desired postoperative pain control, particularly in invasive procedures like extractions. Discrepancies in the timing of LA administration during dental procedures under GA remain. Townsend et al. [21] reported that 79% of pediatric dentists administer LA at the beginning of such cases, while De Verbizer et al. [22] found that only 46% of oral surgeons do so preoperatively [23] Further research is needed in this area to be able to provide standardized guidelines for LA usage in dental surgery treatment under GA.

This study had several limitations. First, the relatively small sample size (*n* = 34, 17 per group) may limit the generalizability of our findings. Although the sample size was calculated based on El Batawi [17], a post hoc power analysis could further confirm the robustness of the results and is recommended for future research. Additionally, the inclusion of only ASA I-II children introduces potential selection bias, and the focus on maxillary teeth may limit the applicability to mandibular procedures, which often require different anesthesia techniques. The exclusion of patients needing extractions may also influence the results. Future studies should include extractions and assess pain management strategies based on treatment type. Further research should also evaluate the effect of LA on intraoperative vital signs and explore deep pain pathways in dental procedures. Expanding the sample size in subsequent studies will be essential to validate these preliminary findings and contribute to evidence-based improvements in pediatric dental care guidelines and practices.

## 5. Conclusion

In conclusion, LA provides better intraoperative vital sign stability, particularly regarding heart rate and ETCO_2_. Also, patients who did not receive LA had statistically significantly higher ETCO_2_ than those patients who received LA. These vitals can be associated with pain intraoperatively; however, though significant, these values provide marginal clinical relevance at this time. With a greater sample size, the trends we are seeing may have clinical implications in future research.

## Figures and Tables

**Table 1:** Characteristics of the two intervention groups (with and without local anesthesia).

Variables	Group LA+(*n* = 17)	Group LA−(*n* = 17)	*p*-Value
Gender, *n* (%) Boys Girls	9 (52.9%) 8 (47.1)	10 (58.8%) 7 (41.2%)	0.73
Age, mean ± SD (years)	4.09 ± 1.38	4.07 ± 1.28	0.959
MAP^a^, mean ± SD (mmHg)	70.82 ± 6.55	68.11 ± 6.43	0.233
MAP^b^, mean ± SD (mmHg)	68.35 ± 6.19	71.52 ± 6.21	0.145
MAP^c^, mean ± SD (mmHg)	68.35 ± 6.19	71.58 ± 6.18	0.137
HR^a^, mean ± SD (bpm)	103.29 ± 16.13	102.76 ± 15.59	0.923
HR^b^, mean ± SD (bpm)	99.05 ± 15.78	109.76 ± 15.6	0.055
HR^c^, mean ± SD (bpm)	98.88 ± 15.76	108 ± 15.31	0.097
SPO_2_^a^, mean ± SD	99.94 ± 0.24	99.82 ± 0.39	0.301
SPO_2_^b^, mean ± SD	99.94 ± 0.24	99.82 ± 0.39	0.301
SPO_2_^c^, mean ± SD	100 ± 0	100 ± 0	NA
ETCO_2_, mean ± SD	36.17 ± 0.46	37.41 ± 1.97	0.046
Aldrete score, mean ± SD	9.47 ± 0.51	9 ± 0	0.001

*Note:* SPO_2_, oxygen saturation; test, independent sample *t*-test.

Abbreviations: ECO_2_, end-tidal carbon dioxide; HR, heart rate; LA, local anesthesia; MAP, mean arterial pressure.

^a^Physiological parameters recorded before the procedure.

^b^Physiological parameters recorded immediately upon entering the pulp chamber.

^c^Physiological parameters recorded after removing the coronal pulp.

**Table 2:** MAP values in the two intervention groups with and without local anesthesia for dental treatment of children under general anesthesia on MAP of the patients.

Variables	Local anesthesia	Mean (mmHg)	Std. deviation	*N*
MAP^a^	Yes	70.8235	6.55968	17
	No	68.1176	6.43120	17

MAP^b^	Yes	68.3529	6.19416	17
	No	71.5294	6.21608	17

MAP^c^	Yes	68.3529	6.19416	17
	No	71.5882	6.18525	17

Abbreviation: MAP, mean arterial pressure.

^a^Physiological parameters recorded before the procedure.

^b^Physiological parameters recorded immediately upon entering the pulp chamber.

^c^Physiological parameters recorded after removing the coronal pulp.

**Table 3:** Mauchly and Greenhouse–Geisser test results for MAP of dental treatment of children under general anesthesia on MAP of the patient.

Effect	Test statistic	Degrees of freedom (df)	*p*-Value
MAP (Mauchly's test)	*W* = 0.026	2	<0.001
MAP (Greenhouse–Geisser)	Epsilon = 0.507	—	—
MAP (Huynh–Feldt)	Epsilon = 0.524	—	—
MAP (lower-bound)	Epsilon = 0.500	—	—

Abbreviation: MAP, mean arterial pressure.

**Table 4:** HR changes in the two study groups of dental treatment of children under general.

Local anesthesia	Type III SS	df	Mean square	*F*	*p*-Value	Partial eta squared
Yes	212.12	2	106.06	138.25	<0.001	0.896
No	450.63	2	225.31	110.29	<0.001	0.873

Abbreviation: HR, heart rate.

## Data Availability

The data that support the findings of this study are available from the corresponding author upon reasonable request.
